# Elements of Physiology, Including Physiological Anatomy

**Published:** 1852-01

**Authors:** 


Art. VIII.—*Elements of Physiology, including Physiological Anatomy.* By William B. Carpenter, M.D., F.R.S., F.G.S., Examiner in Physiology and Comparative Anatomy in the University of London; &c., &c. Second American, from a new and revised London edition. With 190 Illustrations. Philadelphia: Blanchard & Lea. 1851. pp. 566: 8 vo.

This is the third treatise on Physiology from the pen of Dr. Carpenter and the least of the three. His "Principles of Physiology, General and Comparative," an extensive and recondite work, has but lately been introduced to American readers. They are familiar with his admirable "Principles of Human Physiology." The volume before us, though combining in some degree the scope of the other two, cannot be regarded as a mere abridgement of them, having been written for the most part according to the author's own account, with very little reference to them, and with every desire to make it complete in itself. In this effort he has been entirely successful; he has produced a manual as complete as can be found—we will not say in all the literature of physiology—but on any branch of science pertaining to medicine. We do not affirm that in all respects it is equal to its predecessors. It is not so large; it is not so copious in illustration, nor so minute in its details; but on this very account it is better adapted to the wants of the student—a more complete manual.—At the same time that all needless detail has been avoided, it has evidently been the study of its judicious and learned author to avoid the more serious error of sending forth an imperfect and superficial sketch of the science.

"His object has rather been," as he well expresses it himself, "to convey to the student as clear an idea as possible of those principles of physiology, which are based on the broadest and most satisfactory foundation, and to point out the mode in which these principles are applied to the explanation of the phenomena presented by the living actions of the human body. In this manner has the anthor desired to prepare him for that more detailed study of the latter, which becomes necessary when physiology is pursued (as it ought to be) in connexion with the changes produced in the living body by morbific and remedial agents, and is thus taken as a guide in the study of the causes, prevention and treatment of disease—which should be the primary object of attention with every one who undertakes the practice of his profession."

Dr. Carpenter, as our readers generally know, belongs to the school of physiologists, who admit of physical and chemical agencies in the animal economy. He is as far removed as possible from the strait sect of vitalists. He is, perhaps, even ultra in some of his chemical doctrines in regard to certain vital functions. It may be that he is inclined to assimilate the living body too much to the chemist's laboratory. Such, however, is not our belief. On the contrary, we are persuaded that the progress of physiology, in past times, has been seriously retarded by the assumption, that its phenomena were all to be attributed to the operation of a mysterious vital principle, the laws of which we could hardly ever hope to comprehend, or if mastered at all, must be sought for by methods of investigation entirely distinct from those which are employed in other sciences. Vital action is something peculiar, it is true, but its laws are to be attained by the same processes which have proved so successful in advancing physics and chemistry; its phenomena must be collected and compared, and then submitted to the same method of reasoning as that which we use in determining the forces and properties on which other phenomena depend. Such are the principles embraced in the work before us, and upon which modern physiology has been cultivated with so much success.

We are to collect the phenomena, not so much by experiments on living animals, as was the fashion some years ago, as by taking advantage of those "experiments ready prepared by Nature," which we find in the variety of forms of living organized beings peopling the globe. If we would learn the significance of any part or organ, we are not to go about mangling some poor dog or ass in order to determine it, but go to the animals most simple in their structure, and after ascertaining what it is in them, trace it through all the stages of its development until it appears complete in the highest races.

One of the most remarkable generalizations of physiological sciences, that "*every organized fabric*, however complex"—not only "the vast tree, but the feeling, thinking, intelligent man, *has its origin in a cell*—a closed vesicle or minute bag, formed by a membrane in which no definite structure can be discerned, and having a cavity which may contain matters of variable consistence." Of such a cell the whole organism of some simple plants entirely consists: they are made up of it; it is all the plant. Such is that curious growth found sometimes on the snow in some countries, the *Protococcus nivalis* or "red snow," giving to the surface the color of blood. The germ from which the more complex vegetables and even the highest animals spring, "differs in no obvious particular from the permanent condition of one of these lowly beings." The powers of the latter are restricted to a multiplication of new cells, each like the parent; but the former pass through many transformations and a complex fabric with diversified elements is the result. The most highly organized being, however, consists of cells which have undergone no such transformation, each having its particular object in the general economy, and the history of each being essentially the same as if it were maintaining a separate existence.

Let us, with our author, pursue the history of the solitary cell of one of the simplest cryptogamic plants from its first development to its final decay. In the first place, it takes its origin from a *germ,* it may be, a *molecule* too microscopic to be seen without a powerful glass. It increases in size, and a distinction at last appears between its parts which at first seemed homogeneous—between its transparent exterior and its colored interior; and thus we have the first indication of the *cell-wall* and the *cavity.*—The growth continues and this distinction becomes more obvious; the cell-walls are seen to be transparent and homogeneous in texture, whilst the contents of the cavity are distinguished by their color, which is generally green or crimson. They, too, at first appear to be homogeneous, but soon become granular, and these granules grow ultimately into the germs of new cells which are set free by the bursting of the parent cell. This is the end of one generation, but the commencement of the life of a new brood. Each of these germs may become developed into a cell, and in its turn multiply its kind by a similar process.

And this seems to be, briefly, the history of all living beings. In all, the cell originates in a germ or reproductive body, which was prepared by a parent cell. We have no reason to believe that chemical forces can originate the germ of even the humblest plant, but every one to conclude that all vegetables as all animals are the offspring of parents, to which they bear a resemblance in every essential particular. "But how does this *germ,* this apparently homogeneous molecule, become a cell?" We answer that it is endowed with the property of drawing materials to itself from the elements around, and of incorporating these with its own substance. The vegetable cell requires for food only carbonic acid, water and ammonia, and out of these developes new cells, and all the parts of the vegetable fabric. Sugar and starch, gum and albumen result from their combination, and these in one place produce the bark of the plant and in another the wood, in one the coloring matter of the leaves, and in another the fruit—tissues the most diverse in structure and properties thus taking their origin in the same organizable material, the sap or cambium. In like manner, we see in animals from the same circulating fluid all the various elements of the complex organism developed, bone in continuity with bone, muscle with muscle, and nerve with nerve.

In all this there is something to remind us of chemical affinity, and yet much that chemistry cannot imitate or explain. Upon chemical principles we should expect bone to attract to it the elements of bone in the blood, and muscle to attract the fibrin, but there is no refined chemical process by which fibrin can be formed. We have therefore no resort but to say that this, as well as the muscle which it goes to develop, is the product of vitality. We must conclude that there is some peculiar force inherent in living beings by which their various functions are performed. Nay, it is certain that—

"Every kind of cell has its own specific endowments; and generates in its interior a compound peculiar to itself. The nature of this compound is much less dependent upon the nutrient materials which are supplied to the cell, than upon the original inherent powers of the cell itself, derived from its germ. Thus we find that the "red snow" and "gory dew" invariably form a peculiar red secretion; and that they will only grow where they can obtain, from the air and moisture around, the elements of that secretion. Again, the "yeast plant" invariably forms a secretion analogous to animal proteine; and it will only grow in a fluid which supplies it with the materials of that substance. Hence the red snow would not grow in a fermentable saccharine fluid; nor would the yeast plant vegitate on damp cold surfaces. Yet there is little difference, if any, between their cell-walls, in regard to chemical composition. So, also, we shall find hereafter, that one set of cells in the animal body will draw into themselves, during the process of growth, the elements of bile; another, the elements of milk; another, fatty matter; and so on: the peculiar endowments of each being derived from their several germs, which seem to have an attraction for these substances respectively, and which thus draw them together; whilst the cell-wall appears to have a uniform composition in all instances."

Not only is each cell possessed of its own specific endowments but has some distinct office which it is specially adapted to perform. Thus in the act of secretion there are true secreting cells, and parent-cells to develope them as fast as they are destroyed. The parent-cells themselves possess no secreting power, nor do the true secreting cells possess any reproductive power; but the vital forces of the former are entirely expended in the production of the latter, which, having reached maturity and performed their function, die and are cast off. In like manner the cells which constitute the fibrillæ of the muscular fibre have simply the office of contraction to perform, with no power of self-multiplication; and consequently their vital force having been expended in the act of muscular contraction, they perish and are removed.—Their renewal, we have reason to believe, is accomplished by the myolemma, which takes no part in muscular contraction, but is possessed of the reproductive capacity.

"All the truly *vital* phenomena," Dr. Carpenter concludes, "however diversified, are but results of the operation of one and the same force, whose particular manifestations are determined by the nature of the material substratum through which it acts: the same fundamental agency producing simple *growth* in one case, *transformation* in another, *multiplication* in a third, *mechanical movement* in a fourth, whilst in a fifth it developes *nervous power*, which may itself operate in a variety of different modes."

The question then comes up, what is the source of this vital force? Inherently an attribute of the primordial cell, it is nevertheless dependent for its activity upon those external agencies known as vital stimuli. It can only manifest itself when called forth by light and heat.—The plant cannot decompose carbonic acid except with the aid of light, and the energy with which it effects its decomposition is in strict ratio, other things being equal, with the illuminating power of the rays which it receives. And heat is equally indispensable to the growth not only of vegetables but of animals, the activity of the latter being in proportion to the temperature of their bodies.—There is a remarkable correlation between heat, light and electricity which strikingly illustrates the functions of living beings. Thus the motion of a body retarded or resisted by friction, gives rise to heat; and on the other hand, heat applied to any form of matter produces expansion, which is motion. Electricity is developed by the same friction, and electricity retarded gives rise to calorie. Electricity is also excited by the action of heat in a certain combination of metals, and the electrical current excites, not only heat, but light, magnetism, or motion, according to the nature of the substances throughwhich it is transmitted. These forces generate chemical affinity and in turn are generated by it, in all cases a material *substratum* being essential.

The same correlation seems to exist between these physical forces and the vital endowments.

"Thus we may say that light and heat acting upon the organic germ, become transformed into the vital force, in the same manner as heat acting upon a certain combination of metals becomes electricity, or as electricity acting upon iron developes itself as magnetism; and we shall find that this view is in complete harmony with all the phenomena of vital action. Moreover, the vital force thus engendered frequently manifests itself in producing physical or chemical phenomena; thus completing that mutual relationship or correlation, which has been shown to exist among the physical and chemical forces themselves."

We have examples of this principle in the production of heat by animals and certain plants, in the extrication of light by some tribes of the former, and more remarkably in the development of light, heat, magnetism, and electricity by certain species of fish. In the latter instance there is a striking transmutation of the vital into the physical forces, as in other circumstances we have a similar conversion of the physical into the nervous force. But, although electricity is capable of exciting the nerves to a performance of their function, and the nerves in the torpedo or gymnotus electricus produce electricity, we are not therefore to conclude that the forces are identical. There is a striking analogy between them; they stand to each other in the relation that caloric stands to magnetism, or electricity to chemical affinity. The vital forces give rise to physical phenomena, and the physical forces are transmuted, in living beings, into vital activity. Nor is this all; for these physical forces derived from the inorganic world return to it again as the result of vital activity, along with the materials out of which the living structures were produced.

"The plant forms those organic compounds, at the expense of which animal life (as well as its own), is sustained, by the decomposition of carbonic acid, ammonia, and water; and the light, by whose agency alone this process can be effected, may be considered as metamorphosed into the peculiar *affinity*, by which the elements of these compounds are held together. The heat which plants receive, acting through their organized structures as vital force, serves to augment these structures to an almost unlimited extent, and thus to supply new instruments for the agency of light, and for the production of organic compounds. The whole *nisus* of vegetable life may be considered as manifested in this production; and, in effecting it, each organism is not only drawing material, but force, from the universe around it. Supposing that no animals existed to consume these organic compounds, they would be all at last restored to the inorganic condition by spontaneous decay, which would reproduce the carbonic acid, water, and ammonia, from which they were generated. In this decay, however slow, heat and light are given out, in the same amount as when more evidently produced in the ordinary combustive process; and this sometimes occurs even during the life of the plant, whose vital movements, also, may be considered as restoring to the inorganic universe a certain measure of the force they have derived from it under other forms. Thus in making use of the stores of coal which have been prepared for his wants by the luxuriant flora of past ages, man is not only restoring to the atmosphere the carbonic acid, the water, and the ammonia, of the carboniferous period, but is actually reproducing and applying to his own purposes, the light and heat which were operating to produce the growth of vegetation at that remote period in the earth's history."

The views of the author on this interesting subject are still more fully unfolded in the following passage, which cannot fail to arrest the attention of the reader:

"But the organic compounds which the agency of light and heat upon the vegetable structures has produced, are designed for a much higher purpose, than that of being merely given back to the inorganic universe by decay or combustion; and the forces which hold together their elements have a much more exalted destiny. In serving as the food of animals, a part of them become the materials of their organized tissues, and the instruments through which the nervous and muscular forces are developed; whilst another part are applied to sustain the combustive process, by which the heat of the higher classes is maintained quite independently of the external supply of that force. The greater part of the animal kingdom, however, is dependent, like the vegetable, upon the inorganic universe, for the heat which serves as its organizing force; and it is only under the constant influence of this agent, that the operations of growth, development, and maintenance can take place. The animal is not dependent like the plant upon light; and this is obviously because that agent is chiefly concerned in the preliminary operation, by which the organic compounds are generated as the pabulum of the growing tissues; in fact, the embryo within the germinating seed, which, like the animal, is nourished upon matter previously prepared for it, is most rapidly developed in the absence of light, up to the time when, its store being exhausted, its further supplies must be obtained by its own instrumentality. The vital activity of animals, then, may be considered as chiefly sustained by the chemical forces subsisting in their food, which are set free when the elements are reconverted to their original state; and by the heat which they derive from external sources, or from the combustion of a part of their food. These forces may be considered as in a state of continual restoration to the inorganic universe, during the whole life of animals, in the heat, light, electricity, still more in the motion, which they develope; and, after their death, in the production of heat and light during the processes of decay. During animal life, there is a continual restoration to the mineral world, of the carbonic acid, water, and ammonia, which have been appropriated by plants; and it will hereafter appear, that the amount thus given off by the animal organism bears a close correspondence, on the one hand, with its degree of vital activity, as shown in the amount of heat and motion which it generates, and, on the other, with the amount of the organic compounds which it consumes as food. So that, on the whole, there is a strong reason to believe that the entire amount of force (as of materials), received by an animal during a given period, is given back by it during that period, provided that its condition at the end of the term is the same as it was at first; and further, that all the force (like the material), which has been expended in the building up of the organism, is given back by its decay after death."

Thus, we have seen, it is by the agency of cells that all the functions alluded to are performed. The muscles contract, digestion, nutrition, secretion are accomplished by means of cells. The cell in nutrition, as in muscular contraction, is worn out in the performance of its function and cast off, so that the process by which the fabric is built up involves decay, and food is required to reproduce the cells wasted in it.

In like manner, the reproduction of animal and vegetable species is performed by cells. This function has been esteemed the most mysterious of all that pertain to the higher animals; but, as remarked by Dr. Carpenter, we do not see that there is anything more difficult to understand in the fact, "that the parent-cell organizes and vitalizes the protoplasma which it has elaborated, so that it should form the germ of a *new* individual possessing similar properties with itself; than in its incorporating the same material with *its own* structure, and causing it to take a share in its own actions." It consists essentially in the preparation of a cell-germ, which, when set free, gradually develops itself into a structure like that from which it sprang. And these reproductive cell-germs are formed, like the tissue and the contents of the parent-cell, from the nutrient materials which it has the power of bringing together and combining.

In the higher animals re-production requires the union of the sexes; but in some of the lower tribes both the male and the female germ is furnished by the same individual; and, as showing the close relation of the generative function to that of nutrition, it may be mentioned that the *sex* of the plant seems to be dependent, in some cases, upon the circumstances attending its growth, particularly the temperature. Melons and cucumbers, for example, it has been found by experiment, will produce none but male or staminiferous flowers, if their vegetation be accelerated by heat; and all female or pistilliferous flowers, if its progress be retarded by cold. And something of the same modification is wrought in the bee by food; not, indeed, a change of sex, but an arrest of development in the sexual organs of the female by which *neuters* are produced. In every hive of bees, it is known to the naturalist, the majority of individuals consists of these neuters, having the organs of the female sex undeveloped, and are therefore incapable of re-production; that function being restricted to the *queen*, who is the only perfect female in the community. "If by any accident the queen be destroyed, or if she be purposely removed for the sake of experiment, the bees choose two or three from among the neuter eggs that have been deposited in their appropriate cells, which they have the power of converting into queens The first operation is to change the cells in which they lie into *royal* cells, which differ from them considerably in form, and are of much larger dimensions; and when the eggs are hatched, the maggot is supplied with food of a very different nature from the farina or bee-bread, which has been stored up for the nourishment of the workers, being of a jelly-like consistence, and pungent stimulating character. And after the usual transformations the grub becomes a pefect queen, differing from the neuter bee into which it would otherwise have changed, not only in the development of the reproductive system, but in the general form of the body, the proportionate length of the wings, the shape of the tongue, jaws, and sting, the absence of the hollows on the thighs in which the pollen is carried, and the loss of the power of secreting wax."

In the former illustration, the sex was determined by the amount of heat stimulating the vital processes; in the latter, the development of the sexual apparatus depends upon the *kind of food* supplied. Both show how closely allied are the nutritive and reproductive functions.

We have seen that there is a certain ratio between the duration of life and vital activity—that, in a word, the former is inversely proportioned to the latter. Those tissues in which life is most vigorous are the tissues in which change is most rapid; and the animals most active in their habits are those which perish most speedily if not duly supplied with food. The principle is well exhibited in plants, in which we see the active organs of nutrition, the leaves, perishable and transitory, while the stem undergoes but little change when once completely formed. The most striking manifestation, however, of this connexion between vital activity and the duration of life is afforded by what has been termed *dormant vitality*—a condition in which, without any appreciable amount of life or change, an organized structure may reman unaltered for centuries, and then go through its regular series of vital operations, as if these had never been interrupted. This state, as remarked by Dr. Carpenter, differs from life, because life is a state of activity; and it differs quite as widely from death, because death implies not merely a suspension of activity, but a total loss of vital properties. In this state, the vital properties remain; but they are prevented from manifesting themselves by the want of the necessary conditions.

"Of this dormant vitality it may be well to adduce some examples; which may assist in impressing on the mind of the student the general views here put forth. This condition is manifested in the most remarkable manner by the seeds and germs of plants; many of which are adapted to remain, for an unlimited period, in a state of perfect repose, and yet to vegetate with the greatest activity, as soon as ever they meet with the necessary conditions.—Thus the sporules of the fungi, which can only develop themselves in decaying organized matter, seem universally diffused through the atmosphere, and ready to vegetate with the most extraordinary rapidity, whenever a fitting opportunity presents itself. This at least appears to be the only feasible mode of explaining their appearance, in the forms of mould, mildew, &c., on all moist decaying substances; and that there is no improbability in the supposition itself, is shown by the excessive multiplication of these germs, a *single individual* producing not less than *ten millions* of them, so minute as when collected to be scarcely visible to the naked eye, rather resembling thin smoke, and so light as to be wafted by every movement of the atmosphere; so that, in fact, it is difficult to imagine any place from which they can be excluded. Moreover, it is certain that an equally tenacious vitality exists in the seeds of higher plants. Those of most species inhabiting temperate climates are adapted to remain dormant during the winter; and may be easily preserved, in dry air, and at a moderate temperature, for many years. Some of those which had been kept in the Herbarium of Tournefort during upwards of a century, were found to have preserved their fertility. Cases are of no unfrequent occurrence, in which ground that has been turned up, spontaneously produces plants dissimilar to any in their neighborhood. There is no doubt that in some of these cases, the seed is conveyed by the wind, and becomes developed in spots which afford congenial soil, as already remarked in the case of the fungi. Thus it is commonly observed that clover makes its appearance on soils which have been rendered alkaline by lime, by strewed wood-ashes, or by the burning of weeds. But there are many authentic facts, which can only be explained upon the supposition, that the seeds of the newly-appearing plants have lain for a long period imbedded in the soil, at such a distance from the surface as to prevent the access of the air and moisture; and that, retaining their vitality under these circumstances, they have been excited to germination when at last exposed to the requisite conditions.—Thus Professor Lindley states as a fact, that he has raised three raspberry-plants from seeds taken from the stomach of a man, whose skeleton was found thirty feet below the surface of the earth, at the bottom of a barrow which was opened near Dorchester; as his body had been buried with some coins of the Emperor Hadrian, there could be no doubt that the seeds were 1600 or 1700 years old. Again, there are undoubted instances of the germination of grains of wheat, which were enclosed in the wrappers of Egyptian mummies, perhaps twice that number of years ago; the wheat being different in some of its characters from that now growing in the country."

Vital activity, not life itself, is dependent upon external stimuli. Life cannot manifest itself without these agencies, but it is not originated by them. The act of organization and the consequent development of peculiar properties in the tissues which are produced by it, can only be attributed to the vital force of a pre-existing organism. The phenomena called forth from organic beings by the operation of physical forces, are never seen when these forces operate upon inorganic matter. We call the principle which stirs in living beings the vital force, and we are as ignorant of its essential nature as we are of the nature of gravitation, electricity, or chemical attraction; but we assume that it is a *peculiar* principle, because its phenomena are unlike those of any other. It is not caloric, nor electricity, nor the force which causes chemical union among elementary bodies; it differs as widely from them as they do from one another. But just as heat acting under certain conditions may be transformed into electricity, and electricity under other conditions, may be converted into heat, so may either of these forces, acting under conditions which an organized fabric alone can supply, be converted into vital force, whilst, in their turn, as we have seen, they may be generated by the nervous system of animals. The torpedo can at the same time impart the electrical shock, deflect the magnetic needle, emit a spark, develop heat, and decompose chemical compounds.

Of the nature of this power, as we have said, we know nothing; we are led by our consciousness to refer it to a divine will.

"So also, if we endeavor to assign a cause for the existence of a cell-germ, we are led at first to fix upon the vital operations of the parental organism by which it was produced; and for these we can assign no other cause than the peculiar endowments of *its* original germ, brought into activity by the forces which have operated upon it. Thus we are obliged to go backwards in idea from one generation to another; and when at last brought to a stand by the origin of the race, we are obliged to rest in the divine will as the source of those wonderful properties, by which the first germ developed the first organism of that race from materials previously unorganized, this organism producing a second germ, the second germ a second organism, and so on without limit, by the uniform repetition of the same processes. Yet we are not to suppose that the *continuation* of the race is really in any way less dependent upon the will of the Creator, than the *origin* of it. For while science leads us to discard the idea that the Deity is continually *interfering*, to change the working of the system He has made,—since it every where presents us with the idea of uniformity in the plan, and of constancy in the execution of it,—it equally discourages the notion entertained by some, that the creation of matter, endowed with certain properties, and therefore subject to certain actions, was the *final* act of the Deity, as far as the present system of things is concerned, instead of being the *mere commencement* of his operations. If it be admitted that matter owes its origin and properties to the Deity, or, in other words, that its *first existence* was but an expression of the divine will, what is its *continued existence*, but a continued operation of the same will? To suppose that it could continue to exist, and to perform its various actions, *by itself*, is at once to assume the property of *self-existence* as belonging to matter, add thus to do away with the necessity of a Creator altogether; a conclusion to which it may be safely affirmed that no ordinarily constituted man can arrive, who reasons upon the indications of mind in the phenomena of Nature, in the same way as he does in regard to the creations of human art."

We venture to believe, that no reader has followed us through this analysis of the first chapter of Dr. Carpenter's work without becoming interested in the subject, and feeling a curiosity to know more of its contents. Its sound and novel philosophy, its rich fund of facts, its clear, logical arrangement, and its polished and elegant style render it one of the most attractive volumes that has for a long time come under our eye. We need hardly add, after all this, that we cordially recommend it to our readers.

